# Genetic Variations Associated with Sleep Disorders in Patients with Schizophrenia: A Systematic Review

**DOI:** 10.3390/medicines5020027

**Published:** 2018-03-24

**Authors:** Konstantinos Assimakopoulos, Katerina Karaivazoglou, Maria Skokou, Marina Kalogeropoulou, Panagiotis Kolios, Philippos Gourzis, George P. Patrinos, Evangelia Eirini Tsermpini

**Affiliations:** 1 Department of Psychiatry, School of Medicine, University of Patras, Rion, Patras 26504, Greece; kassima@upatras.gr (K.A.); karaivaz@hotmail.com (K.K.); maskokou@upatras.gr (M.S.); pgourzis@upatras.gr (P.G.); 2 Department of Pharmacy, School of Health Sciences, University of Patras, Rion, Patras 26404, Greece; mkalo@windowslive.com (M.K.); pkolios7@gmail.com (P.K.); gpatrinos@upatras.gr (G.P.P.); 3 Department of Pathology, College of Medicine and Health Sciences, United Arab Emirates University, Al-Ain, P.O.Box 17666, UAE

**Keywords:** schizophrenia, sleep disorders, genes, polymorphisms, insomnia, circadian, somnolence, restless-legs syndrome

## Abstract

**Background:** Schizophrenic patients commonly suffer from sleep disorders which are associated with acute disease severity, worsening prognoses and a poorer quality of life. Research is attempting to disentangle the complex interplay between schizophrenia and sleep disturbances by focusing not only on demographic and clinical characteristics, but also on the identification of genetic factors. **Methods:** Here, we performed a systematic literature review on the topic of genetic variations in sleep-disordered schizophrenic patients in an attempt to identify high quality investigations reporting scientifically sound and clinically useful data. For this purpose, we conducted a thorough search of PubMed, ScienceDirect and GoogleScholar databases, according to the guidelines of the Preferred Reporting Items for Systematic Reviews and Meta-analysis (PRISMA) protocol. **Results:** Our search yielded 11 eligible studies. Certain genetic variations were reported to be associated with schizophrenia-related sleep disorders. Antipsychotic-induced restless legs syndrome was linked to polymorphisms located on *CLOCK*, *BTBD9*, *GNB3*, and *TH* genes, clozapine-induced somnolence was correlated with polymorphisms of *HNMT* gene, while insomnia was associated with variants of the *MTNR1* gene. **Conclusions:** There are significant genetic associations between schizophrenia and co-morbid sleep disorders, implicating the circadian system, dopamine and histamine metabolism and signal transduction pathways.

## 1. Introduction

Sleep disorders are extremely prevalent among diagnosed schizophrenics, affecting almost 80% of patients [1]. They constitute a source of clinical concern since they are associated with greater symptom severity, increased relapse rates, worse prognoses, and a diminished quality of life [2,3]. In this context, there have been several studies focusing on the epidemiology, clinical characteristics, and pathogenic mechanisms underlying schizophrenia-related sleep disturbances [4,5,6]. Their findings implicate either the exogenous effects of psychoactive agents (psychotropic medications, alcohol, illegal substances) or the endogenous effects of disease-related pathophysiological processes on sleep continuity and architecture. However, most studies agree that sleep disturbances do not constitute a mere epiphenomenon [7], but rather, represent an integral part of schizophrenia’s pathogenesis which is causally linked to the emergence or the precipitation of psychotic symptoms [8,9,10].

Given that both schizophrenia and sleep disorders have been linked to dysfunction in specific neural circuits including dopaminergic and serotoninergic pathways [10], and that both are characterized by a strong genetic component with shared genetic loci [11,12], research is increasingly focusing on detecting genetic variations which are associated with their co-occurrence. While relevant investigations provide evidence regarding the existence of specific gene polymorphisms in sleep-disordered schizophrenic patients, these studies are characterized by great variability in terms of sleep disorder classification and candidate genes. More specifically, sleep disorders constitute a constellation of heterogeneous conditions with unique epidemiological, clinical, physiological and genetic features [13]. Schizophrenic patients report various patterns of disordered sleep, mainly insomnia, restless legs syndrome (RLS) and obstructive sleep apnea syndrome [10]. For this reason, a comparison of studies will not lead to solid conclusions unless it is based on an integrated and analytic approach. A similar approach to the interpretation of genetic findings is also required, given that modern advances in molecular biology have led to the mapping of a significant number of genes which have been implicated in schizophrenia and sleep pathophysiology [12,14]. Reported findings would therefore provide more clinically relevant information if they could be clustered and summarized in a way that would link them to distinct patterns of neurophysiological and intracellular pathways.

In this context, we have conducted a systematic review of studies searching for genetic variations which are associated with sleep disorders in schizophrenic patients. Our primary aim was to integrate existing findings into a comprehensive set of evidence-based knowledge. Furthermore, we sought to detect areas of scientific ambiguity and define directions for future research. To our knowledge this is the first systematic review focusing on this topic.

## 2. Materials and Methods 

A systematic literature search was conducted from three biomedical literature databases, namely PubMed, ScienceDirect and Google Scholar, according to the guidelines of the Preferred Reporting Items for Systematic Reviews and Meta-analysis (PRISMA) protocol [15]. In addition, we performed a manual search using all references of the selected articles in order to detect any additional relevant literature. No chronological restriction was applied to our search strategy. PubMed search was performed using the following terms: (sleep disorders OR restless leg syndrome OR hypersomnia OR insomnia OR parasomnias OR circadian dysfunction) and (schizophrenia OR psychotic disorder OR psychosis OR neuroleptics side-effects). GoogleScholar search was conducted using the following terms: “sleep disorders” or “insomnia” or “circadian function” and “schizophrenia” or “psychosis” or “psychotic disorders”, while ScienceDirect search was performed with the terms “schizophrenia and sleep disorders and genetic variations”. Our search focused exclusively on full papers that encompassed a detailed description of their methodology and findings. Two reviewers (K.K. and E.E.T.) independently reviewed all titles and abstracts retrieved from the search according to specific predefined inclusion criteria. At the next step of the search, both reviewers met to discuss each article’s eligibility and in 85% of cases they managed to reach a consensus. In the remaining cases of unresolved disagreement between the two reviewers, a third reviewer (G.P.P.), blind to the other reviewers’ suggestions, made the final decision.

### Inclusion and Exclusion Criteria

All original studies written in English focusing on the detection of genetic variations, including haplotypes, microsatellites, copy number variants (CNVs) and single nucleotide polymorphisms (SNPs) in patients with schizophrenia, schizoaffective or delusional disorder, and who were also suffering from sleep disorders including antipsychotic treatment-induced sleep disturbances, were considered eligible to enter the review. According to the latest edition of the International Classification of Sleep Disorders (ICSD), sleep disorders are classified in seven major categories, namely insomnia disorders, sleep-related breathing disorders, central disorders of hypersomnolence, circadian rhythm sleep-wake disorders, sleep-related movement disorders, parasomnias, and other sleep disorders [13]. We only included studies in which schizophrenia and sleep disorders diagnosis was based on a comprehensive psychiatric assessment and the use of validated instruments (semi-structured interviews or questionnaires) according to the diagnostic criteria of the Diagnostic Statistical Manual (DSM-5) or the International Classification of Diseases (ICD-10) [16,17]. We excluded studies failing to provide adequate demographic, clinical and genetic data for their participants. We also decided to exclude studies of genetic variations in loci known to be implicated in schizophrenia or sleep pathophysiology—such as genome-wide association studies (GWAS), whose methodology did not include the clinical evaluation of both schizophrenia symptoms and sleep patterns—in an attempt to focus exclusively on findings derived from clinical populations. We used a data collection report to extract data for each study, including name of first author, year of study and publication, country, number and demographic data of participants, clinical characteristics of the sample, genotyping method, genes and genetic variations examined, and major outcomes.

Moreover, a network of the relevant genes was generated through the use of Ingenuity Pathway Analysis (IPA) in order to highlight possible correlations. IPA is a web-based software developed by QIAGEN which can identify gene associations, as well as subnetworks and canonical pathways relevant to a specific dataset (QIAGEN Inc., https://www.qiagenbioinformatics.com/products/ingenuity-pathway-analysis). 

## 3. Results

**Figure 1:**
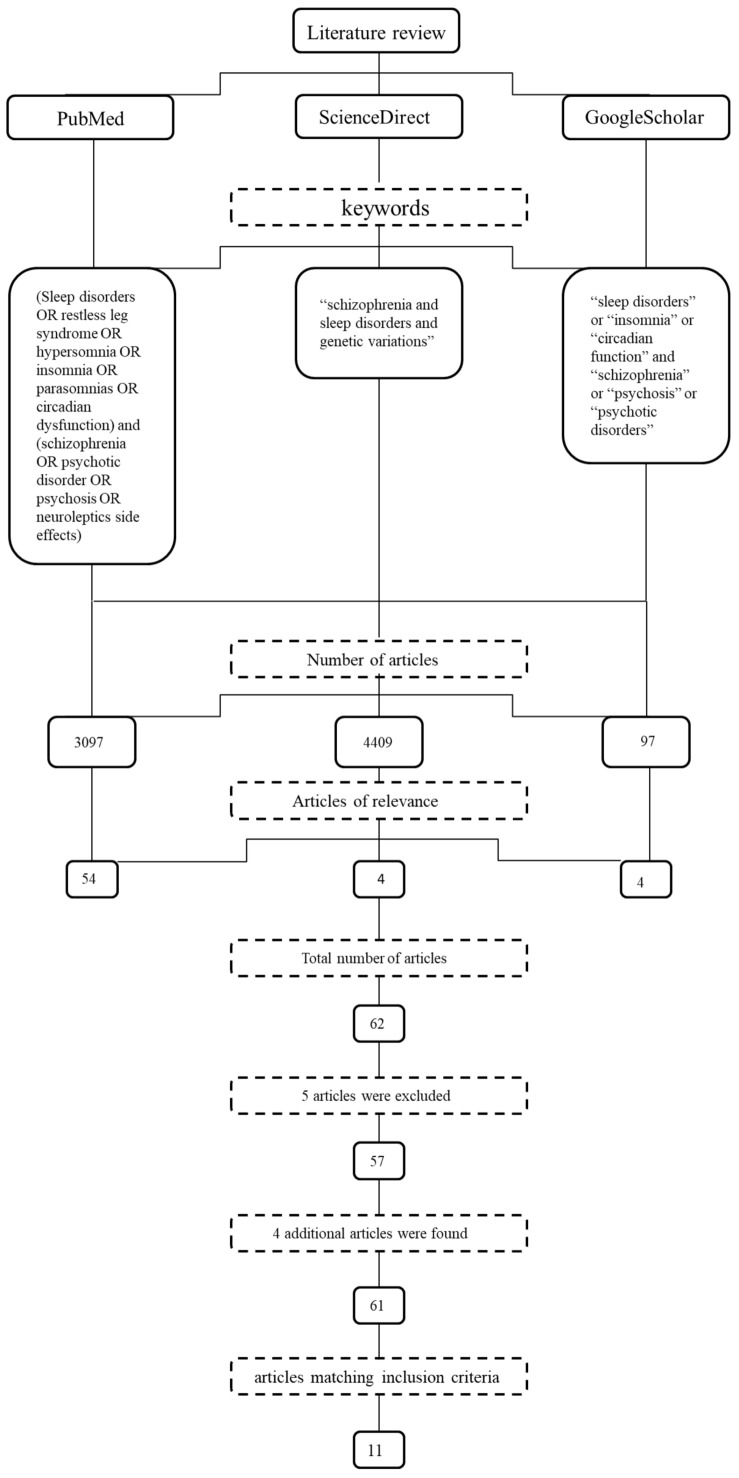
Process of articles selection.

The initial automated search of the databases yielded 3097 titles from PubMed, 4409 titles from ScienceDirect and 97 titles from GoogleScholar. After thoroughly reviewing all abstracts, we concluded that 54 PubMed, 4 ScienceDirect and 4 GoogleScholar articles were of relevance. Among the 62 identified articles, 5 were indexed in more than one database and had to be excluded, leading to a total of 57 original studies as candidates for inclusion in the review. Furthermore, we searched all references lists from these 57 articles in order to detect any unidentified literature; this yielded 4 additional articles, raising the total number of potentially eligible manuscripts to 61. Subsequently in the selection procedure, the full-texts of the 61 articles were carefully and critically evaluated according to the review’s inclusion criteria. This process led to the final selection of 11 studies which fulfilled inclusion criteria and entered the review. It is worthy of note that the 11 selected studies were published in journals with an Impact Factor ranging from 2 to 4 (except for Kang et al. IF = 0.802), Figure 1 depicts a flow diagram analytically describing the process of article selection.

### 3.1. General Characteristics of Studies

All included studies’ general characteristics and major outcomes are summarized in Supplementary Table S1. 

Nine (9) studies were published during the present decade. Eight (8) originated from South Korea and 3 from Europe (2 from Finland and 1 from Spain). Among the 8 articles from S. Korea, 7 reported findings from the same sample of patients. In a similar vein, both articles from Finland reported outcomes derived from the same group of participants. In the Korean studies, participants were exclusively of Asian descent; in the Finnish studies all participants were Caucasian; and in the Spanish study, the authors do not state the racial origin of their sample. In 8 articles, the sample comprised exclusively schizophrenic patients; in 2, the sample included patients with schizophrenia, schizoaffective or delusional disorder; and in 1 study the participants were either schizophrenic patients or healthy controls. As far as types of sleep disorder are concerned, in 7 articles, the authors focused on patients with antipsychotic-induced RLS; in 3 articles the participants suffered from antipsychotic-induced daytime somnolence/sedation; and in 1 study the authors focused on the presence of either insomnia or hypersomnia. In total, in this review we analyzed data derived from 777 patients (of which 39% were females).

The genetic variations of 36 different genes were examined in eligible studies. These genes encode several protein products which include histaminergic, dopaminergic, serotoninergic, glutaminergic and adrenergic receptors (16 genes), enzymes implicated in histaminergic, dopaminergic, noradrenergic and serotoninergic pathways (7 genes), melatonin receptors (2 genes), a protein implicated in limb formation and hematopoiesis (1 gene), proteins implicated in the circadian system (2 genes), enzymes of the cytochrome P450 (3 genes), a protein implicated in ion channels regulation (1 gene), a serotonin transporter (1 gene), a drug transporter (1 gene), a neurotrophic factor (1 gene) and a G-protein (1 gene). Supplementary Table S2 provides data regarding the genes and variations which were found to be significantly associated with the presence of sleep disorders in patients with schizophrenia, including their protein products and the exact pathophysiological pathways in which they are implicated.

### 3.2. Associations between Genetic Variations and Specific Sleep Disorders

According to a series of well-designed studies in a sample of Korean schizophrenic patients on antipsychotic medications, treatment-induced RLS was found to be significantly associated with rs2412646, rs1801260 and rs2412646-rs1801260 haplotype of *CLOCK* gene (Circadian Locomotor Output Cycles Kaput), as well as rs9357271 alleles’ frequency and rs3923809–rs9357271 haplotype of *BTBD9* (BTB Domain Containing 9) gene. Additionally, Cho et al. (2009) genotyped the same sample of patients and found that the rs6356 polymorphism of *TH* gene (Tyrosine Hydroxylase) is associated with increased frequency of RLS only in female subjects [18]. In a similar vein, the probability of antipsychotic-induced RLS was linked to the presence of the C allele of rs5443, of *GNB3* gene (G Protein Subunit Beta 3) [19]. In contrast, in the same group of patients, the authors failed to detect any significant associations between drug-induced RLS and specific polymorphisms of *MEIS1* (Myeloid Ecotropic Viral Integration Site 1 Homolog), *MAOA* (Monoamine Oxidase Type A), *MAOB* (Monoamine Oxidase Type B), *DRD1* (Dopamine Receptor D1), *DRD2* (Dopamine Receptor D2), *DRD3* (Dopamine Receptor D3) and *DRD4* (Dopamine Receptor D4) genes [20,21,22]. Likewise, the frequencies of rs3923809 polymorphism of the *BTBD9* gene, as well as the rs2305160-rs6725296 haplotype of *NPAS2* gene (Neuronal PAS Domain Protein 2), were not significantly different among patients with and without RLS [23,24]. 

In another study with a larger sample of Korean patients and a healthy control group, the presence of insomnia was associated with rs2119882 polymorphism of *MTNR1A* gene (Melatonin Receptor 1A) [25], while hypersomnia did not correlate with any *MTNR1A* polymorphism. 

In a recent study of clozapine treated Finnish patients, rs1455156, rs2737385, rs1050891, rs4245861, rs464633, rs1455158, rs14155157 and rs1050900 polymorphisms on *HNMT* gene (Histamine N-Methyltransferase) were associated with daytime somnolence/sedation. Additionally, a correlation was revealed between clozapine-induced somnolence and rs2737385 polymorphism on *HNMT* gene, rs1552498 and rs17034063 polymorphisms on *HRH1* gene (Histamine Receptor H1), as well as rs697738 polymorphism on *AOC1* gene (Amine Oxidase Copper Containing 1). In a previous study using the same group of participants, TT genotype of rs2470890 polymorphism located on *CYP1A2* gene (Cytochrome P450 Family 1 Subfamily A Member 2), was associated with a significantly greater overall side-effect frequency. However, statistical analysis failed to reveal any significant associations between the above genotype and clozapine-induced daytime somnolence [26]. Likewise, Almoguera et al. (2013) found no significant associations between sleepiness and the variants of several genes which are implicated in risperidone pharmacokinetics and pharmacodynamics, although there was a negligible trend towards a higher frequency of sleepiness in patients carrying the C allele of rs6280 polymorphism, located on *DRD3* gene (dopamine receptor D3) [27].

**Figure 2:**
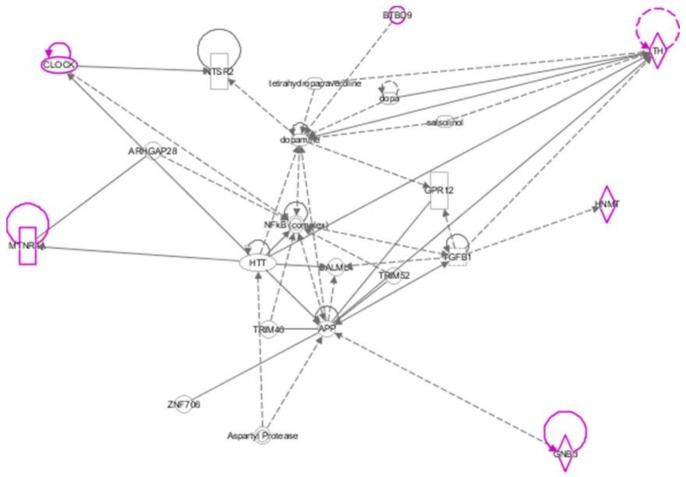
IPA pathway analysis, in which specific genes emerged throughout our review, are shown in purple. As can be seen, GNB3 enhances APP (Amyloid precursor protein) expression, which has been suggested to have growth promoting properties and a role in neuron plasticity. APP is linked with the expression of TGFB1, which encodes a protein that regulates various cell activities including proliferation, differentiation, motility of cells, and apoptosis. TGFB1 is possibly related to an increase in the expression of TH, which is the rate limiting enzyme in the synthesis of dopamine [28]. Tyrosine hydroxylase is a crucial enzyme for converting tyrosine to DOPA in the pathway of the catecholamine biosynthesis. Diurnal expression of TH is mirrored by the diurnal availability of dopamine in the central nervous system. The protein product of the CLOCK gene is a transcription factor with a crucial role in circadian rhythm regulation, which is suggested to be positively linked to the expression of another transcription factor, the protein of the NF-kB gene. The latter also exhibits a central role in circadian and immune mechanisms.

IPA analysis results are shown in Figure 2. Our analysis indicated that *GNB3* participates in a variety of signaling pathways, such as protein kinase A, IL-8, IL-1, Gaq, phospholipase C, Tec kinase, G beta gamma, a-adrenergic, Gas, Gai, opioid, and glutamate receptor signaling, while *MTNR1A* in melatonin, *BTBD9* in dopamine signaling, *HNMT* in histamine degradation, *TH* in protein kinase A and opioid signaling pathway and *CLOCK* in circadian rhythm, adipogenesis and sirtuin signaling pathway.

## 4. Discussion

In conducting the current review, our systematic search of the literature labored over a limited but non-negligible number of studies in order to detect genetic variations in sleep-disordered schizophrenic patients. All included studies possessed a sound and well-elaborated methodological design, however their sample sizes were relatively small thus reducing the statistical value of their conclusions. Moreover, all participants were either of Asian or Caucasian descent, originating from three single geographic regions; in this respect, the existing findings cannot be generalized to other populations with different racial characteristics. To our knowledge, there has been no previous systematic review on this topic, and our findings could constitute an important and novel contribution in the field of schizophrenia and sleep disorders research.

The current review’s major finding was that certain sleep disorders in schizophrenic patients are associated with specific genetic variations. More specifically, in a series of studies conducted in a Korean sample, it was found that antipsychotic-induced RLS is more frequent in patients carrying certain polymorphisms of *CLOCK*, *BTBD9*, *GNB3* and *TH* genes. It should be noted that in all these studies, participants were carefully controlled for a variety of confounding variables, a methodological characteristic which adds further strength to the above findings.

In a similar vein, another research group showed that polymorphisms of *MTRN1* gene (melatonin receptor 1) may be associated with increased frequency of insomnia in patients with schizophrenia. However, a significant limitation of this study was that data analysis did not take into account the type and dosage of antipsychotic medication, and in this respect the observed differences in insomnia prevalence cannot be definitely attributed to differences in genetic variations, but could also be the result of variations in medication dosage.

Furthermore, clozapine-related daytime somnolence was correlated with variants of the *HNMT* gene. Variants of other genes which have been examined did not correlate with the emergence of RLS or somnolence, a finding which could be attributed to the relatively small sample size included in these studies. For this reason, larger-scale studies should be undertaken in order to further address this issue.

It should be noted that the associations we detected involve genes implicated either in the circadian system (*CLOCK* and *MTNR1*) or in signal transduction (*BTBD9* and *GNB3*), and neurotransmitters’ metabolism (*TH* and *HNMT*). The circadian clock is an innate biological system which synchronizes human physiology and behavior with the day/night cycle, thus achieving energy equilibrium and promoting adjustment to environmental demands [29]. Circadian dysregulation has been linked to a variety of physical and mental diseases including sleep disorders and schizophrenia [30]. More specifically, schizophrenic patients exhibit irregular pattern of melatonin secretion which is indicative of a disruption in the circadian rhythmicity of melatonin [27]. In addition, recent studies have reported a loss of circadian expression of certain clock genes, including the CLOCK, in patients with schizophrenia [31,32].

Our review’s findings lend further support to the notion that circadian misalignment is implicated in sleep disturbances of schizophrenic patients and that melatonin plays a crucial role in regulating higher-order neurophysiological processes. However, it remains to be clarified whether this association represents a causal pathophysiological mechanism, or the co-existence of two distinct processes which exert their effect through a common, yet unspecified pathway, for example dopaminergic dysregulation [24,33]. 

Likewise, the detection of a link between schizophrenia-related sleep disorders and genes implicated in neurotransmitter metabolism (*TH* and *HNMT*) and signal transduction (*GNB3* and *BTBD9*) emphasizes the central role of neurotransmission disturbances in the emergence of schizophrenia symptomatology. Tyrosine hydroxylase (TH) is a key-component of dopamine metabolism and has been implicated in schizophrenia, antipsychotic-induced extrapyramidal side-effects and RLS [34,35,36]. Histamine-N-methyltransferase (*HNMT*) is another enzyme which is implicated in neurotransmission by inactivating histamine in the central nervous system [37]. Research has shown that histamine is involved in sleep/wakefulness regulation and its levels demonstrate a circadian rhythmicity [38,39]. Similarly, the G- protein β3 sub-unit (*GNB3*) enhances signal transduction and ion transport [40], and may thus be implicated in dopaminergic and serotoninergic transmission as evidenced by studies linking polymorphisms of *GNB3* gene to depression, antipsychotic medications’ efficacy and neuroleptics-associated weight gain [19,41,42]. Finally, as far as *BTBD9* gene is concerned, it encodes a protein whose exact function has not yet been identified. However, it is broadly expressed in brain structures [43] and it belongs to a family of proteins which regulate ion channels’ tetramerization and gating [44], potentially being involved in inter- and intra-neuronal signal transduction.

It is worthy of note that after applying the Ingenuity Pathway Analysis, it was found that among the six genes which were relevant to out literature review, two of them, *GNB3* and *TH* participate in the same two signaling pathways; Protein Kinase A and Opioid signaling pathways.

Protein kinase A (PKA) is an enzyme, activated by cAMP, which alters the activity of target-proteins through the phosphorylation of specific serine or threonine residues, and regulates a variety of physiological procedures in the nervous system including transcription, synaptic transmission and plasticity, etc. Phosphorylation is a very important procedure, as it can effectively control the activity of proteins and affect their structural, thermodynamic, kinetic and regulatory properties [45].

Opioids receptors are G-protein coupled receptors (GPCR) and are divided into four classes of receptors; mu (MOR), kappa (KOR) delta (DOR), and the nociceptin (NOP) [46]. All of them link to inhibitory G-proteins and their activation can be followed by the initiation of intracellular signaling pathways and, through a variety of cellular processes, can lead to a decreased in nerve impulse transmission and an inhibition of neurotransmitter release [47].

The vast majority of the studies included in this review focused on drug-induced sleep disorders, mainly RLS and somnolence. In this respect it is not clear whether their findings reflect a differential pattern of genetic vulnerability to antipsychotic medications’ side-effects or a distinct schizophrenia endophenotype. Given that sleep disorders may constitute a core component of schizophrenia’s pathophysiology and frequently precede the emergence of full symptomatology [10,48], it would be interesting to assess the relationship between genetic variations and disordered sleep, either in first degree relatives of schizophrenic patients or in high-risk individuals during the prodromal phase, prior to the initiation of any antipsychotic medication. Finally, in all included studies the presence of sleep disorders was assessed with the use of self-reported questionnaires; no research group used a more objective measure of sleep physiology such as polysomnography, actigraphy or EEG recordings, which could provide more detailed information on schizophrenic patients’ sleep quality and architecture [10].

A minor limitation to the current review was that we included studies written exclusively in English and, although the vast majority of biomedical literature is published in the English language, a similar review of non-English literature is also needed in order to ensure that no relevant and clinically useful information evades the scientific community’s attention.

## 5. Conclusions

In conclusion, the current review has reasonably succeeded in identifying and presenting, in a systematic and integrative way, all evidence-based research regarding the association of specific genetic variants with sleep disorders in schizophrenic patients. Retrieved studies were rather limited in sample characteristics and size, indicating that there is a need of larger-scale investigations, i.e., recruiting participants with a broader racial and geographical distribution, in order to further strengthen and expand existing findings. Additionally, our search emphasized the importance of focusing on a wider range of sleep disturbances, as well as on treatment-naïve, high risk populations, in order to capture the full-breadth of the schizophrenia/sleep disorders genetic interplay, and to advance the field.

## Supplementary Materials

The following is available online at http://www.mdpi.com/2305-6320/5/2/27/s1, Table S1: General characteristics and major outcomes of the included studies. Table S2: General characteristics of genes and SNPs whose variations are associated with sleep disorders in patients with schizophrenia.
