# Supplementary material for: Genetic Variations Associated with Sleep Disorders in Patients with Schizophrenia: A Systematic Review
Source: Medicines (Basel). 2018 Mar 24;5(2):27. doi: 10.3390/medicines5020027 (PMC6023503; doi:10.3390/medicines5020027)
Supplement: Supplementary file 1 [file medicines-05-00027-s001.pdf]

Supplementary Materials: Genetic Variations Associated with Sleep Disorders in Patients with Schizophrenia: A Systematic Review

Konstantinos Assimakopoulos, Katerina Karaivazoglou, Maria Skokou, Marina Kalogeropoulou, Panagiotis Kolios, Philippos Gourzis, George P. Patrinos and Evangelia Eirini Tsermpini

Table S1. General characteristics and major outcomes of the included studies.

Author	No ref	Year of publication	Country	No of participants	Demographic characteristics	Clinical characteristics	Method of clinical diagnosis	Genes examined	Genetic variations	Genotyping method	Results
Solismaa	[38]	2017	Finland	176	Gender: 71 (40.3%) females	All participants suffered from schizophrenia, schizoaffective or delusional disorder according to ICD10 and were on clozapine treatment. 75 (42.6%) patients reported moderate or severe daytime somnolence/sedation	ICD10 LUNSERS	*HRH1 HRH2, HRH3, HRH4, HNMT, AOC1* and *HDC*	19 SNPs on *HRH1* (rs1552498, rs17034063, rs6778270, rs7639145, rs17034071, rs443137, rs6773737, rs13061242, rs7619408, rs430353, rs17034093, rs168333, rs6796787, exm-rs1809049, rs1809049, rs1666988, rs6442234, rs11929549, rs346087), 2 SNPs on *HRH2* (rs10079693 and rs2963854), 2 SNPs on *HRH4*	Illumina Infinium Human Core Exome-12 DNA Analysis Beadchip version 1.0	rs1455156, rs2737385, rs1050891, rs4245861, rs464633, rs1455158, rs1455157 and rs1050900 on *HNMT* were associated with sedation<br><br>A trend of association was indicated between rs2737385 (HNMT), rs1552498, rs17034063 (HRH1) and rs697738 (AOC1) with sedation

(rs1421125 and exm1379477), 3 SPs on *HDC* (rs2853766, exm1160903 and rs860526), 12 SNPs on *HNMT* (rs3100702, exm2265208, rs1050900, kgp5104289, rs2737385, kgp3446161, rs4245861, rs4646333, rs1455158, rs1455157, kgp8241224, rs1580111), and 17 SNPs on *AOC1* (rs41465145, rs6977381, rs10952291, rs10893, rs6977081, kgp1103178 rs759008, rs4725373, exm671375, rs12539, rs4725970, rs6980179 rs887588, rs2052129, exm671319, exm671260 and rs4725960)

Kang	[20]	2015	S. Korea	190	Age (mean, SD): 39.6, 9.2 years Gender: 84 (44.2%) females	All participants suffered from schizophrenia according to DSM-IV and were under treatment 96 (50.5%) patients had core symptoms of Restless Leg Syndrome	SCID IV Diagnostic criteria of the IRLSSG	*MEIS1*	*rs*2300478 and *rs*6710341 polymorphis ms	High Resolution Melting Curve Analysis (HRM)	No statistically significant difference in or allele frequency for either rs6710341 or rs2300478
Viikii	[26]	2014	Finland	187	Age (mean, SD): 43.1, 11 years Gender: 70 (39%) females	All participants suffered from schizophrenia, schizoaffective or delusional disorder according to ICD-10	ICD10 LUNSERS	*CYP1A2*	rs2470890	TaqMan SNP Genotyping Assay	rs2470890 (and especially TT genotype) was associated with more severe side effects
Jung	[24]	2014	S. Korea	190	Age (mean, SD): 39.6, 9.2 years	All participants suffered from schizophrenia according to DSM-IV and were under treatment 44 (23.2%) patients fulfilled all criteria for RLS	SCID IV Diagnostic criteria of the IRLSSG	*CLOCK, NPAS2*	*CLOCK:* rs1801260 & rs2412646 & rs1801260-rs2412646 haplotype<br><br>*NPAS2*: rs2305160 & rs6725296 & rs2305160-rs6725296 haplotype	High Resolution Melting Curve Analysis (HRM)	rs2412646 (located on *CLOCK*) was statistically significant between SZ patients with and without RLS, in both allele and genotype level<br><br>rs1801260 (*CLOCK*), rs2305160 & rs6725296 (*NPAS2*) indicated no statistically significant difference between SZ patients with and without RLS, either in allele and in genotype level<br><br>rs1801260-rs2412646 haplotype on *CLOCK* and specifically T-T haplotype appeared to be statistically significant between SZ patients with and without RLS<br><br>rs2305160-rs6725296 haplotype on *NPAS2*, indicated no statistically significant difference SZ patients with and without RLS

Kang	[23]	2013	S. Korea	190	Age (mean, SD): 39.6, 9.2 years<br>Gender: 84 (44.2%) females	All participants suffered from schizophrenia according to DSM-IV and were under treatment<br>44 (23.2%) met the IRLSSG diagnostic criteria and 96 (50.5%) indicated RLS symptoms	SCID IV<br>Diagnostic criteria of the IRLSSG	*BTBD9*	rs9357271, rs3923809 & rs3923809–rs9357271 haplotype	High Resolution Melting Curve Analysis (HRM)	rs9357271 indicated a statistically significant association in allele frequencies between SZ patients with and without RLS and specially T allele was more frequent in SZ patients with RLS<br><br>rs3923809 showed no statistically significant difference in allele frequencies between SZ patients with and without RLS<br><br>rs3923809–rs9357271 haplotype indicated significant association and A-T haplotype was significantly more frequent in SZ patients with RLS
Almoguera	[27]	2013	Spain	111	Age (mean, SD): 45, 13 years<br>49 (44.2%) females	All patients suffered from schizophrenia according to DSM-IV and were on risperidone treatment.<br>66 (56.7%) patients reported somnolence as a treatment side-effect	MINI 4.4<br>UKU Scale	*DRD3*	rs6280	PHARMAChip genotyping array	A non-significant trend was found, between sleepiness and patients carrying the C allele
Park	[25]	2011	S. Korea	289 schizophrenic patients and 505 healthy controls	Schizophrenic patients: 42.9±10.8 years; 100 (34.6%) females<br>Controls: 43.6± 15.5 years; 246 (48.7%) females	118 (40.8%) patients reported insomnia and 34 (11.8%) patients reported hypersomnia	DSM-IV<br>OPCRIT checklist	*MTNR1A*, *MTNR1B*	rs2119882 (*MTNR1A*), rs4753426 (*MTNR1B)*	Sanger Sequencing	rs2119882 was associated with insomnia symptoms of SZ patients, whereas<br>rs4753426 was not

Kang	[21]	2010	S. Korea	190	Age (mean, SD): 39.6, 9.2 years	All participants suffered from schizophrenia according to DSM-IV and were under treatment 44 (23.2%) met the IRLSSG diagnostic criteria and 96 (50.5%) indicated RLS symptoms	SCID IV Diagnostic criteria of the IRLSSG	*MAOA*, *MAOB*	*MAOA*: VNTR 30bp *MAOB*: rs1799836	Electrophoretic Separation	No statistically significant differences
Cho	[18]	2009	S. Korea	190	Age (mean, SD): 39.6, 9.2 years	All participants suffered from schizophrenia according to DSM-IV diagnostic criteria	DSM-IV, Diagnostic criteria of the IRLSSG	*TH*	rs6356	PCR-RFLP (NIaIII)	No significant associations either in allele or genotype level. However, significant differences in alleles and genotypes were detected between female patients with and without RLS
Kang	[22]	2008	S. Korea	190	Age (mean, SD): 39.6, 9.2 years	All participants suffered from schizophrenia according to DSM-IV and were under treatment 44 (23.2%) met the IRLSSG diagnostic criteria, 52 (27.4%) had RLS symptoms but did not meet all IRLSSG diagnostic criteria, and 94 (49.5%) did not have any RLS symptoms	SCID IV Diagnostic criteria of the IRLSSG	*DRD1*, *DRD2*, *DRD3*, *DRD4*	*DRD1*: rs4532 *DRD2*: rs1800497 *DRD3*: rs6280 *DRD4*: rs1800955	PCR-RFLP (rs4532: *Dde*I rs1800497: *Taq*I rs6280: *Msc*I rs1800955: *Fsp*I)	No statistically significant differences

Kang	[19]	2007	S. Korea	178	Age (mean, SD): 39.9, 9.4 years	All participants suffered from schizophrenia according to DSM-IV and were under treatment. 38 (21.3%) of our sample were diagnosed as having the RLS, 50 (28.1%) had RLS symptoms but did not meet all the RLS diagnostic criteria and 90 (50.6%) had no-RLS symptoms	SCID IV Diagnostic criteria of the IRLSSG	*GNB3*	rs5443	PCR-RFLP (*Bse*DI)	No association in genotypic lever, whereas in allelic level there was an association between C allele and increased probability of RLS

Table S2. General characteristics of genes and SNPs whose variations are associated with sleep disorders in patients with schizophrenia. With * is indicated the gene that is not associated with a drug-induced sleep disorder.

Gene	Chromosome	Category	Protein	Function	SNP	Position	Functional Consequence	Alleles	MAF
CLOCK	4	Protein Coding	Circadian Locomotor Output Cycles Kaput	Transcription factor that controls its own and other genes' transcription, collectively known as "clock-controlled genes."	rs2412646	55452605	intron variant, 3 prime UTR variant	T/C	0.39 (T)
					rs1801260	55435202	intron variant, 3 prime UTR variant	A/G	0.23 (G)
					rs2412646-rs1801260 haplotype				
*MTNR1A**	4	Protein Coding	Melatonin Receptor type 1	G protein-coupled receptor which activates multiple intracellular signaling pathways	rs2119882	186555751	upstream variant 2KB	T/C	0.47 (C)
GNB3	12	Protein Coding	G-protein β3 subunit	Couples with membrane receptors forming G protein-coupled receptors and functions as transduction mediator in intracellular signaling pathways	rs5443	6845711	synonymous variant	C/T	0.49 (C)
BTBD9	6	Protein Coding	BTB (POZ) domain-containing 9 protein	Unknown function. Probably implicated in transcription repression, cytoskeleton regulation, tetramerization, and gating of ion channels	rs9357271	38398097	intron variant	T/C	0.44 (T)
					rs3923809-rs9357271 haplotype				
TH	11	Protein Coding	Tyrosine hydroxylase	Catalyzes the conversion of the amino acid L-tyrosine to dihydroxyphenylalanine (DOPA) thus promoting dopamine production	rs6356	2169721	missense variant	C/T	0.43 (T)

HNMT	2	Protein Coding	Histamine N-Methyltransferase	Inactivates histamine by N-methylation. Plays an important role in degrading histamine and in regulating the airway response to histamine.	rs1455156	138016241	intron variant, 3 prime UTR variant	C/A/G/T	0.25 (G)
					rs2737385	138005976	intron variant	T/G	0.27 (G)
					rs1050891	138014190	intron variant, nc transcript variant, 3 prime UTR variant	A/G	0.25 (G)
					rs4245861	138015124	downstream variant 500 b, intron variant and 3 prime UTR variant	C/T	0.25 (T)
					rs464633	143506154	nc transcript variant	A/T	0.50 (A)
					rs1455158	138016068	intron variant, 3 prime UTR variant	C/T	0.36 (T)
					rs1455157	138016182	intron variant, 3 prime UTR variant	T/C	0.36 (C)
					rs1050900	138014348	intron variant, nc transcript variant, 3 prime UTR variant	A/T	0.25 (T)
